# Lumbar Discectomy With Bone-Anchored Annular Closure Device in Patients With Large Annular Defects: One-Year Results

**DOI:** 10.7759/cureus.40195

**Published:** 2023-06-09

**Authors:** Pierce Nunley, K Brandon Strenge, Kade Huntsman, Hyun Bae, Christian DiPaola, R. T Allen, Andrew Shaw, Rick C Sasso, Ali Araghi, Blake Staub, Selby Chen, Laura C Shum, Michael Musacchio

**Affiliations:** 1 Orthopaedics, Spine Institute of Louisiana, Shreveport, USA; 2 Spine Surgery, The Orthopaedic Institute of Western Kentucky, Paducah, USA; 3 Spinal Surgery, Salt Lake Orthopaedic Clinic, Salt Lake City, USA; 4 Orthopaedic Surgery, Cedars-Sinai, Santa Monica, USA; 5 Orthopaedics, UMass Memorial Health, Worcester, USA; 6 Orthopaedics, UC (University of California) San Diego Health System, San Diego, USA; 7 Neurological Surgery, Lyerly Neurosurgery, Jacksonville, USA; 8 Orthopaedic Surgery, Indiana Spine Center, Carmel, USA; 9 Spine Surgery, The CORE Institute, Sun City West, USA; 10 Orthopaedics, Texas Back Institute, Plano, USA; 11 Neurosurgery, Mayo Clinic, Jacksonville, USA; 12 Evidence, Telos Partners, LLC, Milford, USA; 13 Orthopaedics, North Shore University Health System, Evanston, USA

**Keywords:** annular closure device, bone-anchored annular closure device, annular repair, quality of life, spinal surgery, lumbar discectomy, annular defect

## Abstract

Background: Reherniation rates following lumbar discectomy are low for most patients; however, patients with a large defect in the annulus fibrosis have a significantly higher risk of recurrence. Previous results from a randomized controlled trial (RCT) demonstrated that the implantation of a bone-anchored annular closure device (ACD) during discectomy surgery lowered the risk of symptomatic reherniation and reoperation over one year with fewer serious adverse events (SAEs) compared to discectomy alone.

Objective: The objective of this prospective, post-market, historically controlled study was to evaluate the use of an ACD during discectomy, and to confirm the results of the RCT that was used to establish regulatory approval in the United States.

Methods: In this post-market study, all patients (N = 55) received discectomy surgery with a bone-anchored ACD. The comparison population was patients enrolled in the RCT study who had discectomy with an ACD (N = 262) or discectomy alone (N = 272). All other eligibility criteria, surgical technique, device characteristics, and follow-up methodology were comparable between studies. Endpoints included rate of symptomatic reherniation or reoperation, SAEs, and patient-reported measures of disability, pain, and quality of life.

Results: Fifty-five patients received ACD implants at 12 sites between May 2020 and February 2021. In the previous RCT, 272 control patients had discectomy surgery alone (RCT-Control), and 262 patients had discectomy surgery with an ACD implant (RCT-ACD). Baseline characteristics across groups were typical of the overall population undergoing lumbar discectomy. The proportion of patients who experienced reherniation and/or reoperation was significantly lower in the ACD group compared to RCT-ACD and RCT-Control groups (p < 0.05). In the ACD study, the one-year rate of symptomatic reherniation was 3.7%, compared to 8.5% in the RCT-ACD group and 17.0% in the RCT-Control group. In the ACD group, the risk of reoperation was 5.5%, compared to 6.5% in the RCT-ACD group and 12.5% in the RCT-Control group. There were no device-related SAEs or device integrity failures in the ACD, and there were clinically meaningful improvements in patient-reported measures of disability, pain, and quality of life.

Conclusion: In this post-market study of bone-anchored ACD in patients with large annular defects, rates of symptomatic reherniation, reoperation, and SAEs were all low. Compared to the RCT, the post-market ACD study demonstrated lower rates of reherniation and/or reoperation and measures of back pain one-year post-surgery.

## Introduction

Lumbar discectomy for the surgical treatment of disc herniation is an effective procedure for patients with persistent sciatica nonresponsive to conservative care. In this procedure, the defect in the annulus pulposus is typically left unrepaired and this weakened area serves as a potential location for disc reherniation. Following lumbar discectomy, approximately one-third of the patients have a large annular defect and are at high risk for reherniation [1]. Recurrent lumbar disc herniation is reported to occur in 18% of patients after lumbar discectomy [2], and in 27-32% of patients with large annular defects after lumbar discectomy [3,4]. As many as 78% of recurrent cases require additional surgery [3,4], often a high-cost lumbar fusion [5,6]. One large randomized prospective study demonstrated the effectiveness of annular closure in larger defects [7] as a valuable addition to discectomy to reduce rates of recurrent reherniation and the need for subsequent surgery. One year post-surgery is considered the most critical time to predict long-term surgical success, based on rates of reherniation and/or reoperation [8].

Previous results from a large randomized controlled trial (RCT) of 554 patients demonstrated that in patients with large annular defects, the implantation of a bone-anchored device that occludes the annular defect (Barricaid, Intrinsic Therapeutics, Woburn, MA) lowered the risk of symptomatic reherniation and reoperation over one year, with fewer serious adverse events (SAEs), as compared to discectomy alone [9,10]. The current post-market study was designed to evaluate the use of an annular closure device (ACD) during discectomy and confirm the results of the RCT study that was used to establish regulatory approval in the United States.

## Materials and methods

Study design

The design of this post-market study has been previously published [11]. In short, this was a prospective, multicenter, single-arm study enrolling patients at high risk for lumbar disc reherniation based on large annular defects who were treated with limited lumbar discectomy and ACD implantation. The trial is registered at ClinicalTrials.gov (NCT03986580). The protocol was approved by local institutional review boards and the Western Institutional Review Board (Puyallup, WA, United States). All enrolled patients provided written informed consent before study participation.

The study design of the RCT has been previously published [9,12]. Briefly, this was a multicenter randomized trial with the primary objective to determine whether implantation of an ACD during lumbar discectomy reduced the risk of recurrent herniation compared to lumbar discectomy alone. Local ethics review boards approved the study and participants provided written informed consent. The study is registered at ClinicalTrials.gov (NCT01283438).

Patient eligibility criteria, surgical technique, device characteristics, and follow-up methodology including imaging and outcome reporting in this post-market study were comparable to those in the RCT [9,11]. There were no implant design changes between the studies.

Study population

In this post-market study, all patients received discectomy surgery with annular repair. Key eligibility criteria were lumbar disc herniation at L4-L5 or L5-S1, leg pain severity of at least 40 mm on a visual analog scale (VAS) of 100 mm length, a score of at least 40 on the Oswestry Disability Index (ODI) despite at least six weeks of nonsurgical management, and a positive straight leg raise sign on physical examination. Key exclusion criteria were previous spinal surgery at the herniated level, spondylolisthesis with at least 25% slip at the index level, and lumbar osteoporosis. Final eligibility criteria were evaluated intraoperatively by defect size.

The comparison population was patients enrolled in the RCT who had discectomy with an ACD or discectomy alone [9]; only patients with lumbar disc herniation at L4-L5 or L5-S1 were included. All other eligibility criteria between the studies were the same.

Surgical procedure

Patients were treated with limited lumbar discectomy using an interlaminar transflaval approach [13], in which any nucleus that had migrated within the annular defect or beyond the annular wall (including sequestered fragments) was removed. Surgeons were advised to remove loose fragments of the nucleus from within the disc in patients with extrusions or protrusions. At the completion of the limited discectomy procedure, the size of the annular defect was assessed intraoperatively and those defined as large (4 to 6 mm tall and 6 to 10 mm wide) were enrolled in the study and treated with a permanent ACD implant.

The ACD implant for annular closure has a flexible woven polymer fabric component intended to occlude the annular defect and a bone anchor to secure the device to an adjacent vertebral body and prevent migration. After confirmation of the annular defect size, an alignment trial was conducted under fluoroscopic control to establish the correct position and angle of the delivery instrument. The device was then implanted under fluoroscopic guidance.

Outcomes

Follow-up visits occurred at four weeks, three months, and one-year post-index surgery. MRI imaging with axial and sagittal images of the lumbar spine and X-rays were performed during follow-up. Complications, symptomatic reherniation, and reoperation were assessed at each visit. The occurrence of adverse events was evaluated at each visit and adjudicated for seriousness and relation to the procedure or device by an independent data safety monitoring board.

Index level ipsilateral or contralateral symptomatic reherniation was defined as confirmation of reherniation by reoperation, imaging core lab confirmation of reherniation based on MRI performed at an unscheduled visit due to patient symptoms, or imaging core lab confirmation of reherniation based on MRI performed at a scheduled visit in patients with ODI ≥ 40, VAS-Leg ≥ 40, and positive leg raise sign, or with an adverse event deemed related to reherniation, lumbar/leg pain, or a neurological event. This definition for symptomatic reherniation was identical to the one utilized in the RCT study. Index level ipsilateral or contralateral reoperations were defined as any surgical procedure performed at the level of the original herniation, regardless of side or reason, during follow-up. An additional outcome that includes the rate of reherniation and/or reoperation is reported as the composite rate.

Patient-reported measures of disability, pain, and quality of life were also assessed at each follow-up visit. The ODI is a self-administered questionnaire containing 10 sections: pain intensity, personal care, lifting, walking, sitting, standing, sleeping, sex life, social life, and traveling. The scores from each section are totaled and divided by the total possible score to obtain a final percentage of disability, with a higher percentage indicating greater disability [14]. The suggested minimal clinically important difference (MCID) for ODI is between 10 and 14 [15-17]. Leg and back pain VAS scores measure leg and back pain on a 100 mm scale, from 0 (no pain) to 100 (maximum pain) [18]. The suggested MCID for VAS pain scales is between 12 and 19 [15,16]. The EuroQol 5-Dimensions (EQ-5D) score measures the patient's quality of life, translated to an index between −0.109 (worst) and 1.00 (best) based on the US population [19]. The suggested MCID for EQ-5D is 0.2 [17].

Statistical analysis

Baseline characteristics were summarized with descriptive statistics. Baseline differences between groups were determined using one-way ANOVA for continuous variables or the chi-square test for categorical variables. Categorical variables were described with percentages and counts; continuous variables were described with means and standard deviations. Time-to-event data were analyzed using Kaplan-Meier methods. Statistically significant differences in surgical and patient-reported outcomes between groups post-surgery were evaluated using one-way ANOVA, and differences in the proportion of patients requiring reherniation or reoperation were evaluated using Fisher’s exact test, with a p-value < 0.05 representing a statistically significant difference.

## Results

Baseline demographics

**Figure 1:**
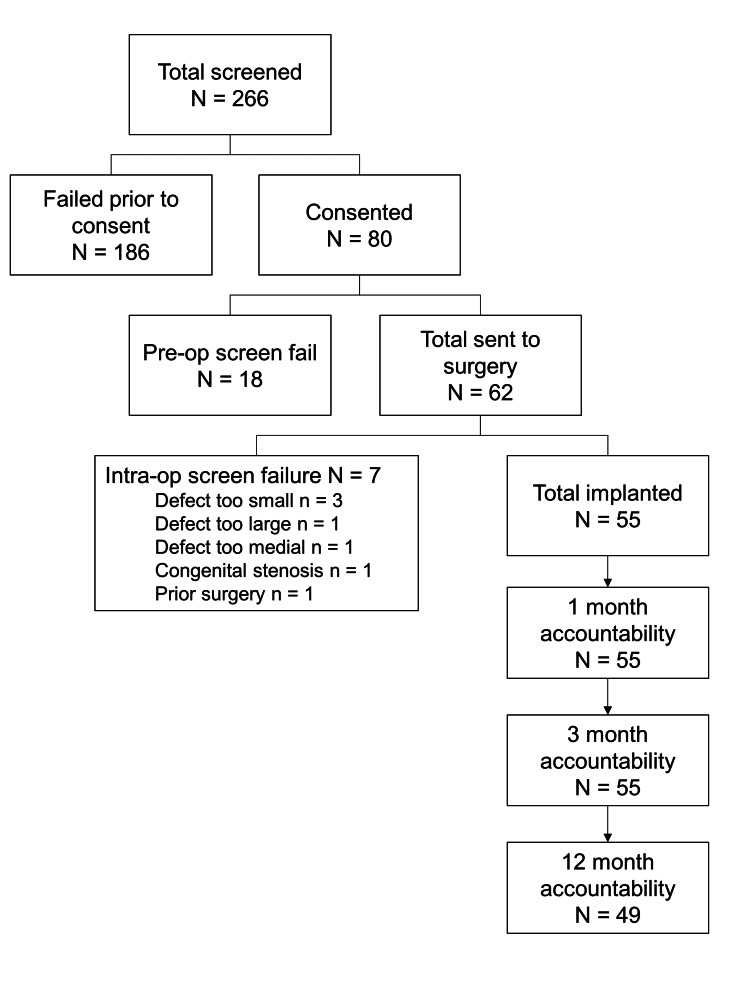
Enrollment and accountability in the post-market study

In the post-market study, 55 patients were enrolled at 12 sites between May 2020 and February 2021 and received ACD implants (Figure 1). A total of 266 patients were screened, of which 204 patients were excluded before surgery, either prior to consent or at preoperative screening. Of the 62 patients sent to surgery, there were seven intraoperative screening exclusions. This resulted in 55 implanted patients with 100% (55/55) follow-up at one and three months and 89% (49/55) at one year. In the RCT, 272 control patients who had discectomy surgery alone (RCT-Control), and 262 patients who had discectomy surgery with an ACD implant were included (RCT-ACD) (Table 1). To match the post-market study participants, only those from the RCT with herniations at L4/L5 and L5/S1 were included in this analysis.

**Table 1:** Baseline demographics ACD: annular closure device; RCT: randomized controlled trial; SD: standard deviation; BMI: body mass index; VAS: visual analog scale; ODI: Oswestry Disability Index; EQ-5D-3L: EuroQol 5-Dimensions 3 Level. * p < 0.05 vs. RCT-ACD; † p < 0.05 vs. RCT-Control.

Characteristics	ACD (N = 55)	RCT-ACD (N = 262)	RCT-Control (N = 272)
Age, mean (SD)	41.1 (12.7)	42.9 (10.8)	43.9 (10.3)
Female sex, n (%)	22 (40.0)	113 (43.1)	105 (38.6)
BMI, kg/m^2^, mean (SD)	28.5 (4.4)*^†^	26.2 (4.1)	26.4 (4.2)
Smoking status, n (%)			
Current	8 (14.5)*^†^	116 (44.2)	121 (44.5)
Ever	24 (43.6)*^†^	167 (63.7)	172 (63.2)
Level of herniation, n (%)			
L4/L5	27 (49.1)	124 (47.3)	101 (37.1)
L5/S1	28 (50.9)	138 (52.7)	171 (62.9)
Annular defect width by size, n (%)			
6 mm	4 (7.3)	47 (17.9)	42 (15.4)
7 mm	11 (20.0)	62 (23.7)	53 (19.5)
8 mm	12 (21.8)	84 (32.1)	83 (30.5)
9 mm	12 (21.8)	36 (13.7)	45 (16.5)
10 mm	16 (29.1)	33 (12.6)	49 (18.0)
VAS-Leg, mean (SD)	83.4 (14.1)	80.8 (15.0)	81.2 (14.4)
VAS-Back, mean (SD)	67 (26.8)*^†^	56.1 (30.3)	56.2 (21.4)
ODI, mean (SD)	58.0 (14.5)	58.8 (12.3)	58.3 (13.8)
EQ-5D-3L, mean (SD)	0.44 (0.21)	-	-

Broad demographics were similar across groups (Table 1). The mean age was 41 years for ACD, 43 years for RCT-ACD, and 44 years for RCT-Control. Across groups, 39-43% were female. More patients from the RCT groups were current or former smokers compared to those from the post-market study. A greater number of patients in the ACD group had larger annular defect widths, as compared to those enrolled in the RCT study; 51% had defects of 9 or 10 mm in the ACD group, as compared to 26% for RCT-ACD and 35% in RCT-Control. Despite larger defect sizes in the post-market study, baseline measures of disability and leg pain were similar across groups; however, back pain was greater in the post-market study. Across groups, baseline characteristics were typical of the population undergoing lumbar discectomy.

Surgical parameters

Surgical parameters are shown in Table 2. Mean (±SD) annular defect width was 8.5 (±1.3) mm in the ACD group, 7.8 (±1.2) mm in the RCT-ACD group, and 8.0 (±1.3) mm in the RCT-Control group. The mean (±SD) nucleus removed was similar across groups: 1.1 (±0.6) cc in the ACD group, 1.3 (±0.9) cc in the RCT-ACD group, and 1.3 (±0.8) cc in the RCT-Control group. Mean (±SD) blood loss was lower in the ACD group: 31.9 (±24.5) cc vs. 69.4 (±32.6) cc in the RCT-ACD group and 65.1 (±85.0) cc in the RCT-Control group (p < 0.05 vs. RCT-ACD and vs. RCT-Control).

**Table 2:** Surgical characteristics ACD: annular closure device; RCT: randomized controlled trial. Values are mean (SD); * p < 0.05 vs. RCT-ACD; ^† ^p < 0.05 vs. RCT-Control.

Parameter	ACD (N = 55)	RCT-ACD (N = 262)	RCT-Control (N = 272)
Estimated blood loss, cc	31.9 (24.5)*^†^	69.4 (32.6)	65.1 (85.0)
Nucleus removed, cc	1.1 (0.6)	1.3 (0.9)	1.3 (0.8)
Annular defect width, mm	8.5 (1.3)*^†^	7.8 (1.2)	8.0 (1.3)

Reherniation and reoperation rates

**Figure 2:**
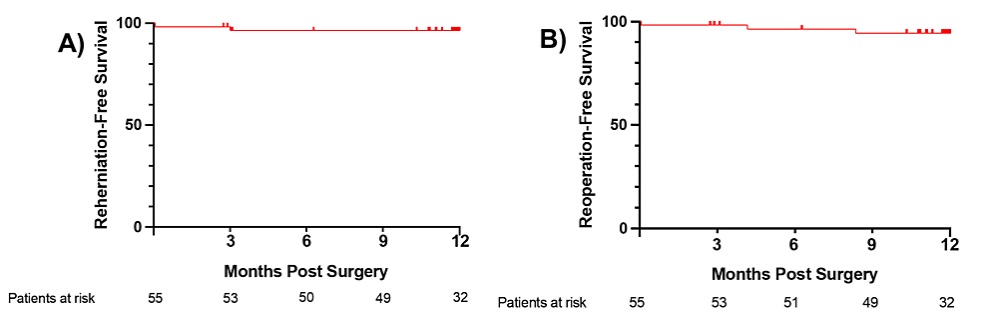
Reherniation (A) and reoperation (B) in post-market study

Symptomatic reherniation and reoperation were rare in the post-market study. Kaplan-Meier curves for reoperation-free survival and reherniation-free survival are shown in Figure 2. Symptomatic reherniation occurred in 3.7% of patients and reoperation occurred in 5.5% of patients (Table 3). Taken together, these patients account for a 7.3% composite rate. The composite rate was significantly lower in the ACD group compared to the RCT-Control (p < 0.05). In comparison, the risk of reoperation was 6.5% in the RCT-ACD group and 12.5% in the RCT-Control group. The risk of experiencing a symptomatic reherniation was 8.5% in the RCT-ACD group and 17.0% in the RCT-Control group.

**Table 3:** One-year risk outcomes ACD: annular closure device; RCT: randomized controlled trial. * p < 0.05 vs. RCT-ACD; † p < 0.05 vs. RCT-Control. Composite rate = reherniation and/or reoperation.

Outcome	ACD (N = 55)	RCT-ACD (N = 262)	RCT-Control (N = 272)
Reherniation	3.7%^†^	8.5%	17.0%
Reoperation	5.5%	6.5%	12.5%
Composite rate	7.3%^†^	10.3%	18.8%

Patient-reported outcomes

**Figure 3:**
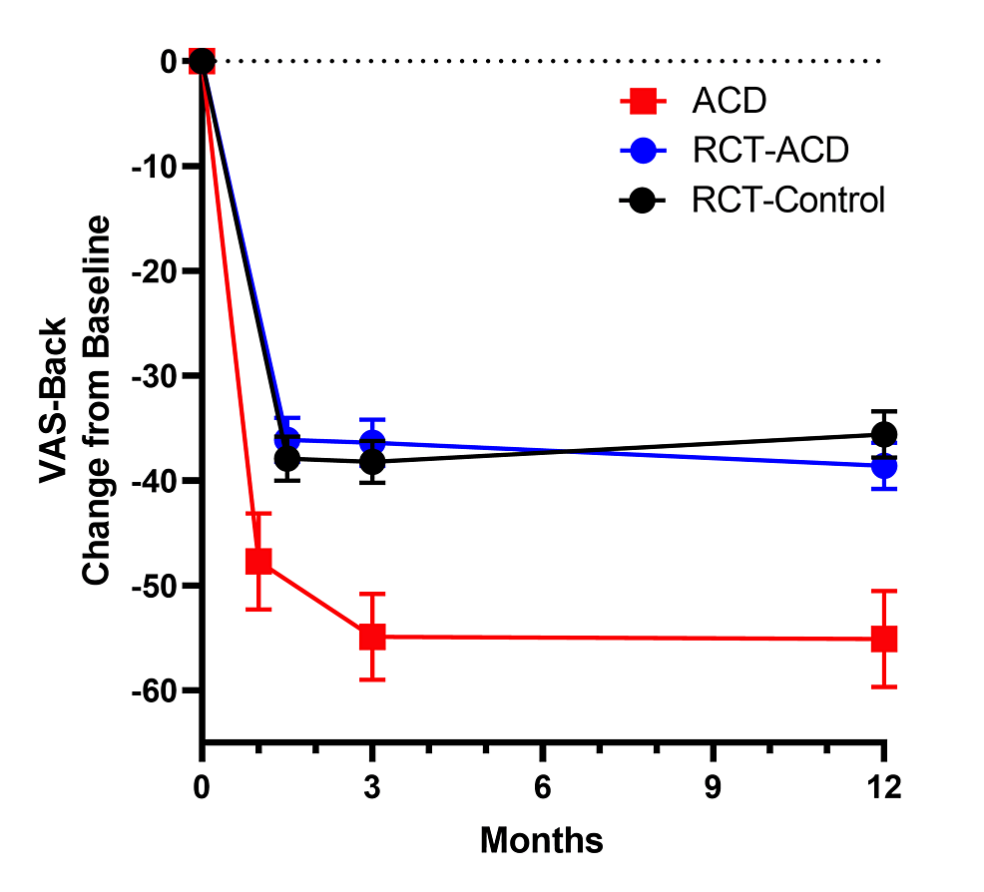
VAS-Back, change from baseline ACD: annular closure device; RCT: randomized controlled trial; VAS, visual analog scale. Values are mean (±SE).

**Figure 4:**
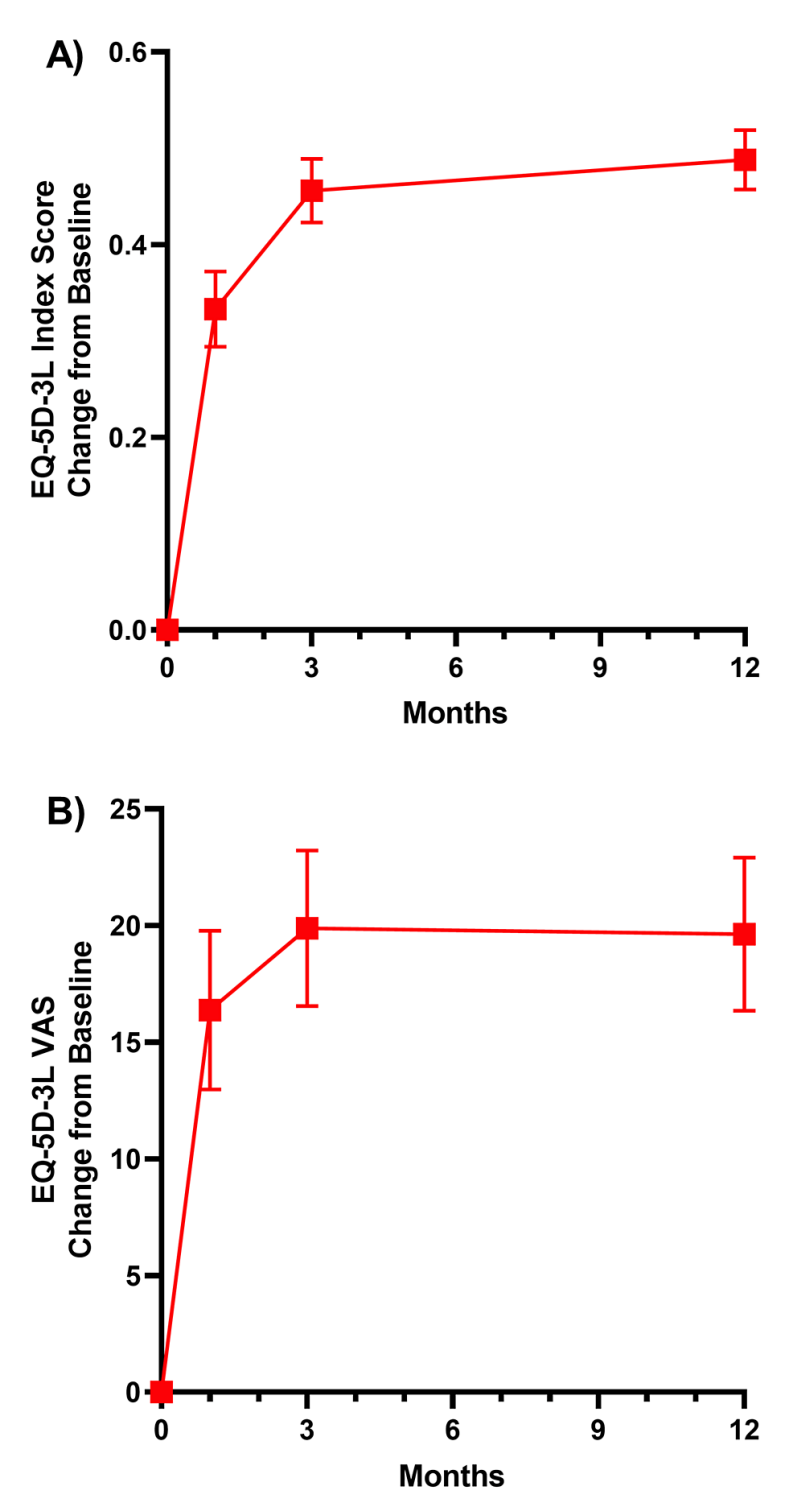
Change from baseline in EQ-5D-3L index (A) and EQ-5D-3L VAS (B) in the post-market ACD study ACD: annular closure device; VAS: visual analog scale; EQ-5D-3L: EuroQol 5-Dimensions 3 Level. Values are mean (±SE).

The mean change from baseline in ODI was similar across groups, with improvements greater than MCID observed at four to six weeks and sustained through one year (Table 4). At one-year post-surgery, the mean change from baseline in leg pain VAS score was -76.7 in the ACD group, -67.2 in the RCT-Control group, and -70.0 in the RCT-ACD group (Table 4), far surpassing the MCID. At three months post-surgery, the mean change from baseline in back pain VAS was -54.9 in the ACD group, significantly improved compared to -38.2 in the RCT-Control group, and -36.4 in the RCT-ACD group (Figure 3). This was sustained at one-year post-surgery. The mean change from baseline was -55.1 in the ACD group, -35.6 in the RCT-Control group, and -38.6 in the RCT-ACD group (p < 0.05 vs. RCT-Control and RCT-ACD) (Figure 3 and Table 4). Quality of life, as measured by EuroQol 5-Dimensions 3 Level (EQ-5D-3L) and VAS scores, increased significantly at every timepoint compared to the baseline in the post-market study (Figure 4).

**Table 4:** Patient-reported outcomes ACD: annular closure device; RCT: randomized controlled trial; VAS: visual analog scale; ODI: Oswestry Disability Index. Values are the mean change from baseline (SD). * p < 0.05 vs. RCT-ACD; † p < 0.05 vs. RCT-Control.

	ACD (N = 55)	RCT-ACD (N = 262)	RCT-Control (N = 272)
VAS-Leg	-76.7 (19.4)	-70.0 (24.8)	-67.2 (24.3)
VAS-Back	-55.1 (32.1)*^†^	-39.5 (34.5)	-35.7 (35.0)
ODI	-48.0 (18.0)	-46.0 (17.8)	-44.1 (18.7)

In the post-market study, of the 41 patients who were working full time before the index surgery, 92.7% (38/41) had returned to work by three months, with a median return-to-work time of two weeks (manuscript in preparation). Of these full-time employees, 35 provided return-to-work data at one-year post-surgery: 97.1% (34/35) had returned to working full time at one year, with a median return-to-work time of 2.5 weeks. Of the two patients who were working part-time before surgery, one had returned to work part-time, and one was working full-time one year after surgery.

Adverse events

No additional device or procedure-related SAEs were reported since the three-month follow-up timepoint. As described by Nunley et al. [20], four SAEs were reported in three subjects (5%). The four SAEs were for hematoma, reherniation, infection, and neurological function decline. In comparison, device or procedure-related SAEs were reported in 14% of RCT-Control subjects and 7% of RCT-ACD subjects through one year.

## Discussion

Previous studies demonstrated that implantation of a bone-anchored ACD during discectomy surgery lowered the risk of symptomatic reherniation and reoperation over one year, with fewer SAEs compared to discectomy alone [9]. The results presented here extend and confirm the findings of the previous RCT and extend the short-term results of the post-market study, which demonstrated clinically meaningful improvements in symptoms and low rates of symptomatic reherniation and reoperation [20]. The ACD group had the highest proportion of patients with the largest annular defect size (10 mm), yet still had outcomes equal to or better than both groups in the RCT, including rates of reherniation and/or reoperation. Additionally, there were no device-related SAEs or device integrity failures.

Reherniation occurs most often within the first year after surgery, demonstrating the importance of this timeframe. One-year rates of reoperation and reherniation in the ACD group were similar to those reported in the RCT-ACD group. Importantly, ACD implantation during discectomy demonstrated benefits without an increase in serious complications, adding to evidence of a favorable risk-benefit profile of this device.

In the post-market study, patient-reported outcomes met the criteria for minimal clinically important differences [15-17] as early as four weeks post-surgery, which were sustained up to one-year post-surgery. This provides valuable information regarding the patient's quality of life and pain measures following ACD implantation. While changes in ODI were similar across groups, statistically significant improvement in back pain scores was observed in the ACD group compared to the RCT-Control. Significant changes in back pain scores were also observed versus the RCT-ACD group. Comparing patient-reported outcomes with published data from lumbar discectomy patients from the Swedish Spine register [21], we see similar trends (N = 7791). Specifically, the change in EQ-5D index score was 0.46 one year post-discectomy in the Swedish Spine register, in line with the results reported here for the ACD group.

The current study was an opportunity to measure and evaluate lessons learned from the RCT. The number of device integrity failures from the RCT study elevated the importance of certain indications and warnings. Whole device migrations from the RCT study (n = 3 in the first year) underlined the need to contraindicate osteopenic patients or those with damaged, weakened, or compromised vertebral bodies in the area of implantation. Similarly, the polymer barrier migrations from the RCT study raised the importance of ensuring that the selected implant size is at least as wide as the annular defect as specified in the instructions for use. There were six implants in the RCT study that were undersized, three of which resulted in polymer barrier migration, reherniation, and reoperation. This underscores the importance of following the sizing guidelines contained in the instructions for use, which include the recommendation to select the larger implant size when possible to provide more coverage and support for the annulus.

Moreover, surgical techniques for precise and reproducible insertion of the anchor have been refined and emphasized in surgical training. This includes making sure the anchor is not rotated and not aiming toward or too close to the endplate, which leads to more accurate placement and a lower chance of failure. These subtle changes are a possible explanation for the lack of device integrity failures in the post-market study and subsequent lower reherniation/reoperation composite rate compared to the RCT study.

This study had several limitations. First, patient-reported outcomes measured at prospectively defined timepoints had the potential to miss acute episodes of a reherniation or reoperation. Second, the single-arm design of this post-market study limits the direct comparison to a control group. However, there are no direct clinical alternatives to the implantation of this ACD to use for comparison in the study, as currently, it is the only FDA-approved method of annular closure. Third, a greater number of patients in the post-market study had larger annular defect widths, as compared to the RCT study; however, this was largely due to preoperative screening in the post-market study being selectively more sensitive for large defects. This could also be considered a potential strength of the study, as these patients had comparable or better outcomes than those in the RCT study. Finally, this study was not powered to determine the noninferiority of outcomes comparing data in the post-market study to the RCT study; however, study design elements and methodology were similar between the post-market and RCT studies, including eligibility criteria, surgical technique, and follow-up. Despite the small study size and the single-arm design, the data presented provide evidence that ACD implantation yields comparable results in multiple settings.

## Conclusions

This post-market study of a bone-anchored ACD in patients with large annular defects validated the results of a previous RCT with low rates of symptomatic reherniation, reoperation, and SAEs. The ACD results were comparable to or better than those observed in the RCT. Clinically meaningful improvements in patient-reported measures of pain and disability were also observed in both studies. In conclusion, rates of symptomatic reherniation and reoperation following lumbar discectomy and ACD implantation were demonstrably low across multiple settings.

## References

[1] Miller LE, McGirt MJ, Garfin SR, Bono CM (2018). Association of annular defect width after lumbar discectomy with risk of symptom recurrence and reoperation: systematic review and meta-analysis of comparative studies. Spine (Phila Pa 1976).

[2] Carragee EJ, Spinnickie AO, Alamin TF, Paragioudakis S (2006). A prospective controlled study of limited versus subtotal posterior discectomy: short-term outcomes in patients with herniated lumbar intervertebral discs and large posterior anular defect. Spine (Phila Pa 1976).

[3] Carragee EJ, Han MY, Suen PW, Kim D (2003). Clinical outcomes after lumbar discectomy for sciatica: the effects of fragment type and anular competence. J Bone Joint Surg Am.

[4] Thomé C, Kuršumovic A, Klassen PD (2021). Effectiveness of an annular closure device to prevent recurrent lumbar disc herniation: a secondary analysis with 5 years of follow-up. JAMA Netw Open.

[5] Ambrossi GL, McGirt MJ, Sciubba DM, Witham TF, Wolinsky JP, Gokaslan ZL, Long DM (2009). Recurrent lumbar disc herniation after single-level lumbar discectomy: incidence and health care cost analysis. Neurosurgery.

[6] Castillo H, Chintapalli RT, Boyajian HH, Cruz SA, Morgan VK, Shi LL, Lee MJ (2019). Lumbar discectomy is associated with higher rates of lumbar fusion. Spine J.

[7] Bailey A, Araghi A, Blumenthal S, Huffmon GV (2013). Prospective, multicenter, randomized, controlled study of anular repair in lumbar discectomy: two-year follow-up. Spine (Phila Pa 1976).

[8] Suri P, Pearson AM, Zhao W, Lurie JD, Scherer EA, Morgan TS, Weinstein JN (2017). Pain recurrence after discectomy for symptomatic lumbar disc herniation. Spine (Phila Pa 1976).

[9] van den Brink W, Flüh C, Miller LE, Klassen PD, Bostelmann R (2019). Lumbar disc reherniation prevention with a bone-anchored annular closure device: 1-year results of a randomized trial. Medicine (Baltimore).

[10] Thomé C, Klassen PD, Bouma GJ (2018). Annular closure in lumbar microdiscectomy for prevention of reherniation: a randomized clinical trial. Spine J.

[11] Strenge KB, DiPaola CP, Miller LE, Hill CP, Whitmore RG (2019). Multicenter study of lumbar discectomy with Barricaid annular closure device for prevention of lumbar disc reherniation in US patients: a historically controlled post-market study protocol. Medicine (Baltimore).

[12] Klassen PD, Hes R, Buoma GJ (2016). A multicenter, prospective, randomized study protocol to demonstrate the superiority of a bone-anchored prosthesis for anular closure used in conjunction with limited discectomy to limited discectomy alone for primary lumbar disc herniation. Int J Clin Trials.

[13] Spengler DM (1982). Lumbar discectomy. Results with limited disc excision and selective foraminotomy. Spine (Phila Pa 1976).

[14] Fairbank JC, Couper J, Davies JB, O'Brien JP (1980). The Oswestry low back pain disability questionnaire. Physiotherapy.

[15] Hägg O, Fritzell P, Nordwall A (2003). The clinical importance of changes in outcome scores after treatment for chronic low back pain. Eur Spine J.

[16] Copay AG, Glassman SD, Subach BR, Berven S, Schuler TC, Carreon LY (2008). Minimum clinically important difference in lumbar spine surgery patients: a choice of methods using the Oswestry Disability Index, Medical Outcomes Study questionnaire Short Form 36, and pain scales. Spine J.

[17] Asher AL, Kerezoudis P, Mummaneni PV (2018). Defining the minimum clinically important difference for grade I degenerative lumbar spondylolisthesis: insights from the Quality Outcomes Database. Neurosurg Focus.

[18] Zanoli G, Strömqvist B, Jönsson B (2001). Visual analog scales for interpretation of back and leg pain intensity in patients operated for degenerative lumbar spine disorders. Spine (Phila Pa 1976).

[19] Burström K, Johannesson M, Diderichsen F (2001). Swedish population health-related quality of life results using the EQ-5D. Qual Life Res.

[20] Nunley P, Strenge KB, Huntsman K (2021). Lumbar discectomy with Barricaid device implantation in patients at high risk of reherniation: initial results from a postmarket study. Cureus.

[21] Elkan P, Lagerbäck T, Möller H, Gerdhem P (2018). Response rate does not affect patient-reported outcome after lumbar discectomy. Eur Spine J.

